# miRNA sequencing analysis of healthy and atretic follicles of chickens revealed that miR-30a-5p inhibits granulosa cell death via targeting Beclin1

**DOI:** 10.1186/s40104-022-00697-0

**Published:** 2022-04-12

**Authors:** Haorong He, Dongmei Li, Yongtong Tian, Qinyao Wei, Felix Kwame Amevor, Congjiao Sun, Chunlin Yu, Chaowu Yang, Huarui Du, Xiaosong Jiang, Menggen Ma, Can Cui, Zhichao Zhang, Kai Tian, Yao Zhang, Qing Zhu, Huadong Yin

**Affiliations:** 1 Farm Animal Genetic Resources Exploration and Innovation Key Laboratory of Sichuan Province, Sichuan Agricultural University, Chengdu, 611130 Sichuan China; 2 National Engineering Laboratory for Animal Breeding and Key Laboratory of Animal Genetics, Breeding and Reproduction, Ministry of Agriculture and Rural Affairs, College of Animal Science and Technology, China Agricultural University, Beijing, 100193 China; 3 Animal Breeding and Genetics key Laboratory of Sichuan Province, Sichuan Animal Science Academy, Chengdu, 610066 China; 4 College of Resources, Sichuan Agricultural University, Chengdu, 611130 China

**Keywords:** Apoptosis, Autophagy, Beclin1, Chicken granulosa cells, MiR-30a-5p, Oxidative stress, RNA-seq, Steroid hormone

## Abstract

**Background:**

The egg production performance of chickens is affected by many factors, including genetics, nutrition and environmental conditions. These factors all play a role in egg production by affecting the development of follicles. MicroRNAs (miRNAs) are important non-coding RNAs that regulate biological processes by targeting genes or other non-coding RNAs after transcription. In the animal reproduction process, miRNA is known to affect the development and atresia of follicles by regulating apoptosis and autophagy of granulosa cells (GCs).

**Results:**

In this study, we identified potential miRNAs in the atretic follicles of broody chickens and unatretic follicles of healthy chickens. We identified gga-miR-30a-5p in 50 differentially expressed miRNAs and found that gga-miR-30a-5p played a regulatory role in the development of chicken follicles. The function of miR-30a-5p was explored through the transfection test of miR-30a-5p inhibitor and miR-30a-5p mimics. In the study, we used qPCR, western blot and flow cytometry to detect granulosa cell apoptosis, autophagy and steroid hormone synthesis. Confocal microscopy and transmission electron microscopy are used for the observation of autophagolysosomes. The levels of estradiol (E2), progesterone (P4), malondialdehyde (MDA) and superoxide dismutase (SOD) were detected by ELISA. The results showed that miR-30a-5p showed a negative effect on autophagy and apoptosis of granulosa cells, and also contributed in steroid hormones and reactive oxygen species (ROS) production. In addition, the results obtained from the biosynthesis and dual luciferase experiments showed that Beclin1 was the target gene of miR-30a-5p. The rescue experiment conducted further confirmed that Beclin1 belongs to the miR-30a-5p regulatory pathway.

**Conclusions:**

In summary, after deep miRNA sequencing on healthy and atretic follicles, the results indicated that miR-30a-5p inhibits granulosa cell death by inhibiting Beclin1.

**Supplementary Information:**

The online version contains supplementary material available at 10.1186/s40104-022-00697-0.

## Introduction

In modern and intensive animal husbandry, the fertility of animals defines its productivity. Chicken eggs are mostly used for breeding next generations and also serve as protein source for human diets. The growth and development of the ovarian follicle correlates with egg production rate in chickens. The follicles develop continuously and ovulate through periodic selection, whereas the unselected follicles disappear from the ovary by processes involved in follicle atresia. Follicular atresia is a normal process in the ovary to regulate the number of follicles in the developing pool. Mostly, few follicles can successfully develop into pre-ovulation follicle before ovulation. Approximately 99% of the follicles will be subjected to atresia during the follicle developmental process [1, 2]. The process of follicle selection directly affects the reproductive performance of female chickens. However, ovarian diseases, such as premature ovarian failure, polycystic ovary syndrome (PCOS) and granulosa cell carcinoma are mainly caused by disorders of atresia [3]. Broodiness in poultry species terminates ovulation which is manifested as atrophy and atresia of ovarian follicles [4]. Previous studies have shown that apoptosis of granulosa cell cause follicular atresia in both mammals and poultry [5, 6]. Usually, follicular atresia occurs when the proportion of apoptotic granulosa cells in the developing follicle reaches more than 10% [7]. Moreover, ovarian follicular atresia in chicken *STAR*ts in the granular layer and it is regulated by apoptosis [8].

Follicle development in chickens is a complex biological process regulated by endocrine hormones that are synthesized by the hypothalamus-pituitary-gonad axis (HPG axis) and various paracrine or autocrine factors in the follicles [9]. In addition, the spatiotemporal expression of a large number of functional genes and the molecular regulation of various transcriptional factors also play vital roles in follicle development. Several studies used high-throughput sequencing technology and bioinformatics to study the functional roles of miRNAs in animals, and they found that miRNAs are involved in regulating follicle development. For instance, miRNA (20-25 nt in length) inhibits gene translation through a 7 nt seed sequence. A single miRNA can target multiple genes to enable few miRNAs to regulate approximately one-third of the coding genes in the entire genome. Furthermore, miRNAs can serve as a “bridge” for other non-coding RNAs (long non-coding RNA and circular RNA) to perform regulatory functions. Long non-coding RNAs and circular RNAs can alleviate inhibitory effects caused by miRNA through adsorbing those miRNAs. This indicates that miRNAs can occupy key positions in the complex regulatory network of non-coding RNAs [10, 11].

In this study, we collected healthy and atretic follicles from laying chickens and broody chickens. The healthy and atretic follicles were ued as two models. Thereafter, we performed miRNA transcriptome sequencing to explore the effects of miRNA on chicken follicle development and atresia. Studies have reported on miRNA transcriptome sequencing in other animal species [12, 13], however, the specific roles and the involvement of miRNAs in chicken ovarian follicular development and atresia is not clear. In this study, after the bioinformatics analysis of the RNA-seq data, we found that miR-30a-5p was differentially expressed in the healthy follicles and atretic follicles. Furthermore, we verified the regulatory function and molecular mechanism of miR-30a-5p in chicken primary granulosa cell in vitro.

## Materials and methods

### Animal and ethics standards

All animal experiments in this study were performed to meet the requirements of animal welfare. It has been approved by Sichuan Agricultural University Institutional Animal Care and Use Committee with the approval number 2019102013. Tianfu broilers chickens (provided by the Poultry Breeding Unit of Sichuan Agricultural University, China) were the chicken breed used in this study.

### Sample collection and total RNA extraction

The follicles used for the deep miRNA sequencing were collected from 200-day-old laying Tianfu broilers and Tianfu Broody Broilers. Three follicular tissues were collected from three chickens per group. The healthy prehierarchical follciles were collected from the laying Tianfu broilers breeders and the atretic prehierarchical follicles were collected from the broody Tianfu broilers breeders. The egg yolk was squeeze off from the granulosa layer and was washed gently with phosphate buffered saline (PBS, Hyclone, Logan, UT, USA). Thereafter, total RNA was extracted using TRIzol reagent (Invitrogen, Carlsbad, CA, USA) according to the manufacturer’s instructions. All the RNA samples extracted were stored at − 80 °C for further analysis. The heart, liver, spleen, lung, kidney, breast muscle, leg muscle, brain, fat, intestine, gizzard, ovary, and uterus tissues were also collected from the chickens for further analysis.

### miRNA and mRNA deep sequencing

We construsted cDNA library from the total RNA using Small RNA Sample Pre Kit (Illumina, Beverly, MA, USA). Thereafter, preliminary quantification was performed using Qubit2.0 (Thermo, Waltham, MA, USA) and then the library was diluted to 1 ng/μL. Then, the quality of the library was determined by the size of the library using Agilent 2100 (Agilent, Santa Clara, CA, USA). Afterwards, we performed miRNA and mRNA deep sequencing using the Illumina novaseq6000 (Illumina). Refer to the Gallus_gallus-5.0 (Ensembl release 94) genome for sequencing data analysis. The expression levels of the miRNAs and mRNAs in each tissue sample were statistically analyzed, and the expression levels were normalized using TPM and FPKM algorithm. The TPM normalization formula is as follows:

$$ TPM=\frac{R\mathrm{eadcount}\times \mathrm{1,000,000}}{M\mathrm{apped}\ r\mathrm{eads}} $$

### Differentially expressed miRNA analysis and target gene prediction

We used DESeq2 [14] based on the negative binomial distribution to analyze the differential expression of the two sets of miRNAs. From the fold change and the corrected significance level (*P* value), two levels were evaluated and the differential miRNAs were screened. The default differential miRNA screening condition was: *P* < 0.05. Each comparison combination were given a differential miRNA set, and the union of all the comparison combinations of the differential miRNA sets in each of the experimental group/sample TPM value were used for hierarchical clustering analysis. After obtaining the differentially expressed miRNA between the groups, miRDB, TargetScan and Diana tools were used to predict the target genes of the differential miRNAs.

According to the correspondence between the miRNAs and their target genes, we performed Gene Ontology and KEGG (Kyoto Encyclopedia of Genes and Genomes) enrichment analyses on each set of differentially expressed miRNA target genes. The GO enrichment analytical method used was GOseq. In this study, we used KOBAS software to detect the statistical enrichment of the candidate target genes in the KEGG pathway.

### Cell culture and transfection

The granular layer of the follicles were separated according to Gilbert’s method [15]. The granular layer was cut into pieces and digested with collagenase type II (BIOFROXX, Einhausen, Germany) after which is was filtered through a 70 μm cell sieve, and then resuspend and plated with Medium 199 (m199, Gibco, Langley, OK, USA), Fetal Bovine Serum (FBS, Gibco) and penicillin-streptomycin (Solarbio, Beijing, China). It was then incubated at constant temperature of 37 °C, 5% CO_2_ and saturated humidity.

The RNA oligonucleotides and overexpression plasmids were designed according to the sequences of miR-30a-5p (miRBase accession number: MIMAT0001135) and Beclin1 (NCBI accession number: NM_001006332.1). Among them, miR-30a-5p inhibitor, inhibitor Negative Control (inhibitor NC), miR-30a-3p mimic, and mimic Negative Control (mimic NC) were constructed using GenePharma (GenePharma, Shanghai, China). The Beclin1 overexpression plasmid was constructed using Tsingke Biotechnology (Tsingke, Beijing, China). Lipofectamine 3000 (Invitrogen) and Opti-MEM (Gibco) were used to transfect the RNA oligonucleotides and overexpression plasmids into the cells.

### Real-time quantitative PCR, western blot and immunofluorescence

The transfected cells were collected and total RNA was extracted. Then the mRNA was reverse transcripted using Prime Script RT Master Mix Perfect Real Time (Takara, Dalian, China), whereas miRNA was reverse transcripted using the one-step miRNA cDNA synthesis kit (HaiGene, Ha’erbin, China). Primers used for the real-time qPCR were designed using Premier 6 software (PREMIER Biosoft, San Francisco, CA, USA). The specific information of the primers is shown in Additional file 3: Table S1. The real-time qPCR was performed in triplicate per sample. The specific reaction included 1 μL cDNA, 0.5 μL reverse primer, 0.5 μL forward primer, 3 μL double distilled water and 5 μL TB Green™ Premix Ex Taq™ II (Takara).

Total protein extraction kit (BestBio, Shanghai, China) and the BCA protein quantification kit (BestBio) were used for the extraction and quantification of transfected cell proteins. The Western Blot and Immunofluorescence were performed following the methods described previously [16]. In addition, BeyoECL *STAR* (Beyotime, Shanghai, China) was to chemically reacted with horseradish peroxidase (HRP) coupled with the secondary antibody to detect and take photograph of the Western Blot strips. The antibodies used in this study are shown in Additional file 4: Table S2. β-Tubulin was used as the loading control. Image J (National Institutes of Health, Bethesda, Maryland, USA) was used to count the gray values of the Western Blot strips. The immunofluorescence images were captured using fluorescence microscope (Olympus, Melville, NY, USA).

### miRNA targeting site mapping and dual luciferase report analysis

After detecting that Beclin1 is the target gene for miR-30a-5p, rnahybrid (https://bibiserv.cebitec.uni-bielefeld.de/rnahybrid/) was used to verify the target relationship according to the sequences of miR-30a-5p and Beclin1, and then the picture of the target mechanisms was drawn.

The Beclin1 3’UTR sequence was inserted into the pcDNA3.1(+) vector by ligase to construct a wild-type or mutant plasmid. Furthermore, the dual luciferase reporter gene was constructed and synthesized using Tsingke Biotechnology (Tsingke), and then it was co-transfected with the RNA oligonucleotides into the granulosa cells. The luciferase activity was detected using Dual-GLO Luciferase Detection System Kit (Promega, Madison, WI, USA) and the luminescence activity of the firefly luciferase and Renilla was also detected using a multifunctional microplate reader (Biotek, Winooski, VT, USA) following the manufacturer’s instructions.

### ELISA analysis

We determined the concentrations of the reproductive hormones such as PRL, LH, and FSH in the serum, and the levels of E2, P4, SOD and MDA in the cell supernatant using chicken ELISA kits (MEIMIAN, Yancheng, Jiangsu) following the manufacturer’s instructions.

### Confocal microscope and transmission electron microscopy

Mcherry-EGFP-LC3 adenovirus was purchased from Hanbio (Hanbio, Shanghai, China). Specific methods followed were previously described [17]. GCs were cultured on the cell slides in a six-well plates and transfected with miR-30a-5p mimic and mimic NC or miR-30a-5p inhibitor and inhibitor NC. Thereafter, they were transfected with adenovirus and observed under a confocal microscope (Olympus, Melville, New York, USA). Similarly, after transfection with the miR-30a-5p inhibitor and inhibitor NC, the granular cells were fixed and then the transmission electron microscopy was performed following the procedures described previously [18]. Finally, the observation was made using JEM-1400 TEM (JEOL, Tokyo, Japan) and the pictures were taken using CCD camera AMT (Sony, Tokyo, Japan).

### Flow cytometry analysis

The cell apoptosis was determined using the flow cytometry. 100 μL binding buffer was added to the transfected granulosa cells to resuspend the cells. Then, 5 μL Annexin v-fitc (Invitrogen, Carlsbad, CA, USA) was added, after staining with fluorescent dye for 10 min in a dark room temperature. Subsequently, 10 μL of PI (BD Pharmingen, Santiago, CA, USA) was added and stained for 5 min. After avoiding light at room temperature, 400 μL of mixed buffer suspension was added and immediately tested on the machine. Thereafter, CytoFLEX flow cytometer (Beckman Coulter, Brea, CA, USA) and Kaluza 2.1 software (Beckman Coulter) were used for detection and data analysis respectively. In addition, ROS was measured using DCFH-DA and the fluorescent probe was also detected using JC-1 to measure the potential of the mitochondrial membrane.

### Statistical analysis

SPSS 19.0 statistical software (SPSS, Inc., Chicago, Illinois, USA) was used for the statistical analysis of the data. There were three biological replicates for each experiment. The unpaired Student’s *t-*test was used for comparative analysis between the two groups. One-way ANOVA analysis was used for the comparative analysis of the multiple groups. At *P* < 0.05, the difference of all statistical tests is considered significant and at *P* < 0.01 was considered extremely significant. All data are shown as least squares mean ± standard error of mean (SEM).

## Results

### Comparison of the phenotypic characteristics and serum hormones between the laying and broody chickens

**Fig. 1:**
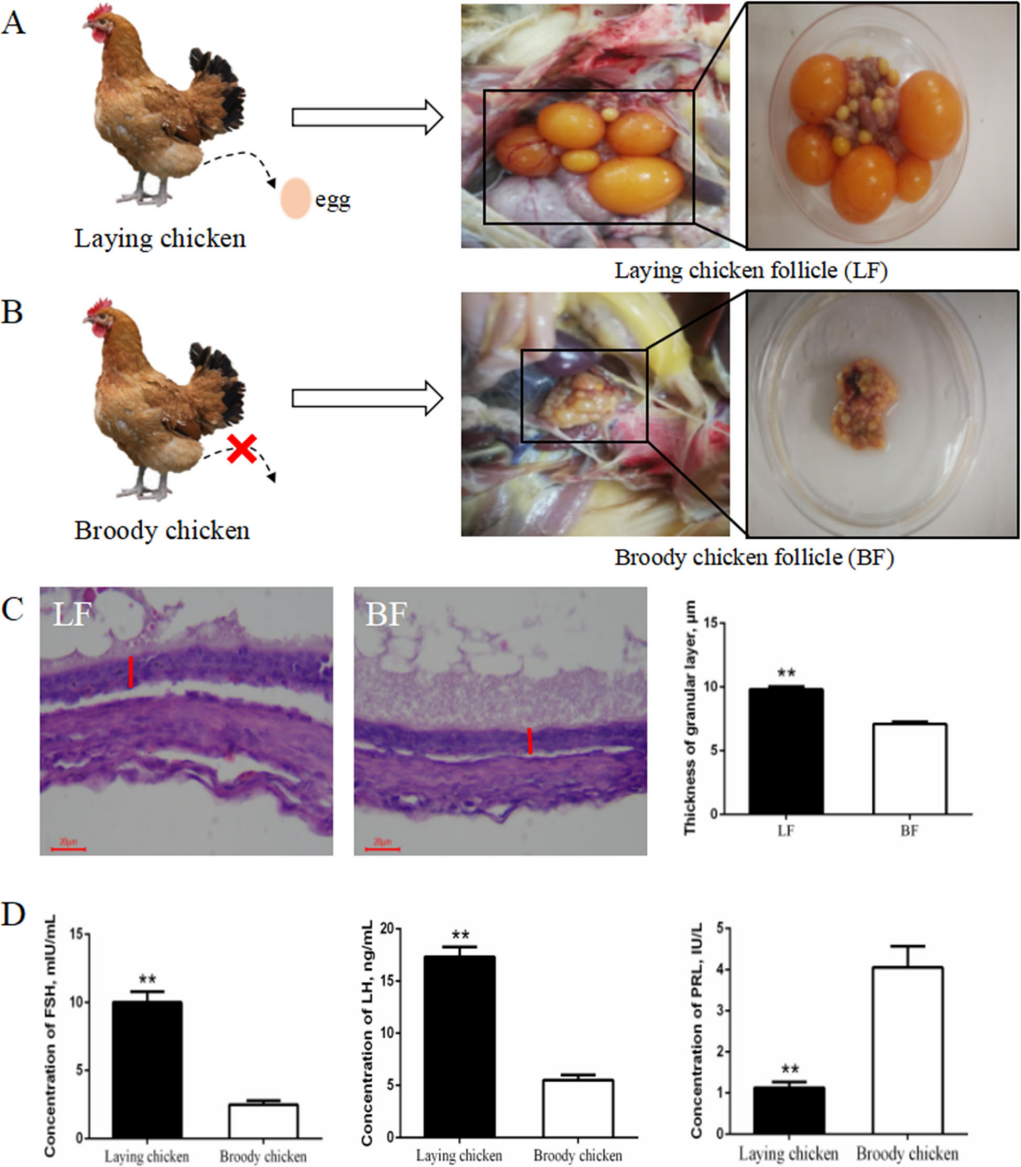
Comparison of morphology, histological characteristics and plasma hormone levels. (**A**, **B**) Morphological characteristics of the healthy follicles and atretic follicles. (**C**) Hematoxylin-eosin staining of the paraffin sections of the healthy follicles and atretic follicles. The red straight line represent the granular layer, and the thickness of healthy follicles and atretic follicles have been measured, μm (*n =* 9). (**D**) Serum hormone levels in the laying chicken and broody chicken (*n =* 3). **P < 0.01. Data are presented as mean ± SEM

We compared the phenotypic characteristics and serum hormones of the laying and broody chicken before tissue sampling. We found that there were follicles at different developmental stages in the ovaries of the healthy sexually mature chicken, including pre-hierarchical follicles and hierarchical follicles. However, in the broody chicken, the ovaries were atrophied, and the follicles were all atretic (Fig. 1A and B). The results of the hematoxylin-eosin staining of the pre-hierarchical follicles showed that the thickness of the follicles (healthy follicle) obtained from the laying chicken chicken was thicker compared to that of the follicles (atretic follicle) obtained from the broody hens (Fig. 1C, *P* < 0.01). Furthermore, we found that the levels of serum luteinizing hormone (LH) and follicle-stimulating hormone (FSH) increased significantly in the laying chicken compared to that of the broody chickens. However, the PRL levels decreased significantly in the laying chicken compared to that of the broody chickens (Fig. 1D, *P* < 0.01).

### Overview of the miRNA RNA-seq data

**Fig. 2:**
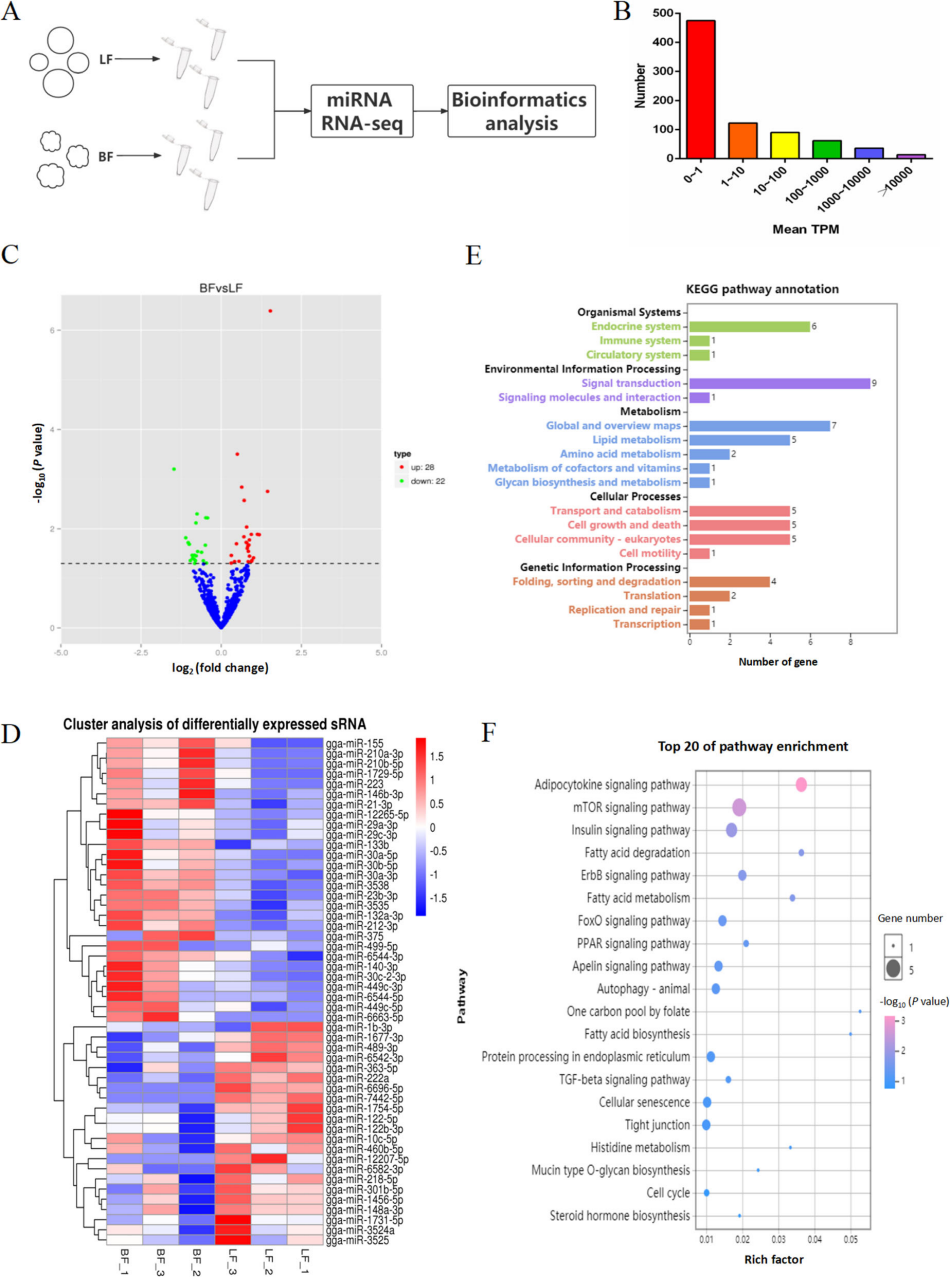
Overview of the miRNA RNA-seq Data. (**A**) Schematic diagram of miRNA sequencing of healthy follicles and atretic follicles. (**B**) Distribution of miRNAs with 0 to > 10,000 mean transcripts per million. (**C**) Volcano map of differentially expressed miRNA. (**D**) Clustering heat map of differentially expressed miRNAs. (**E**,**F**) GO and KEGG analyses of the target genes of differentially expressed miRNA

In this study, we performed deep miRNA sequencing analysis on both healthy and atretic follicles obtained from three chickens each per group, thus, normal laying chickens and broody chickens (Fig. 2A). All the miRNA sequencing data are deposited in the SRA database with the accession number PRJNA721929. We obtained 6 sets of raw reads, ranging from 10,401,755 to 14,548,548. The sequencing quality values Q20 and Q30 were all greater than 98% and 94% respectively. After the low-quality reads with connectors were removed, the clean reads obtained were greater than 97% (Additional file 5: Table S3). The above reads were mapped to the specified reference range sequence in the miRBase (https://www.mirbase.org/) [19]. We obtained 730 known miRNAs by comparison and 70 unknown miRNAs were discovered. Among these miRNAs, those with an average > 100 transcripts per million (TPM) were clasified as the less abaundant, whereas those with an average < 1 TPM were classified most abundant (Fig. 2B). In addition, the value of TPM is negatively correlated with the number of miRNAs, indicating that only a few miRNAs affect follicular development (Additional file 1: Fig. S1). A total of 50 differentially expressed miRNAs (DEMs) were identified among the known miRNAs of which 22 miRNAs were highly expressed in the healthy follicles compared to the atretic follicles, whereas the other 28 miRNAs were down-regulated in the healthy follicles compared to the atretic follicles (Fig. 2C). Cluster analysis showed that miRNA expression patterns were different in healthy follicle and atretic follicle (Fig. 2D). Furthermore, we performed Gene Ontology and KEGG enrichment analysis on the target genes predicted by DEM. The Gene Ontology enrichment analysis results showed that the main enrichment of target genes in bioengineering were involved in cell process, cell proliferation, developmental processes and reproduction (Additional file 2: Fig. S2). KEGG enrichment analysis of 50 differentially expressed miRNA predicted target genes mainly involved in biological functions in the endocrine system, immune system and circulatory system, whereas those related to cellular processes were involved in cell growth and death, transportation and catabolism. We also observed that the target genes were mainly enriched in cell autophagy, steroid hormone biosynthesis, mTOR signaling pathway, insulin signaling pathway, TGF-β signaling pathway, and FoxO signaling pathway (Fig. 2E and F).

### Analysis of differentially expressed miRNAs

**Fig. 3:**
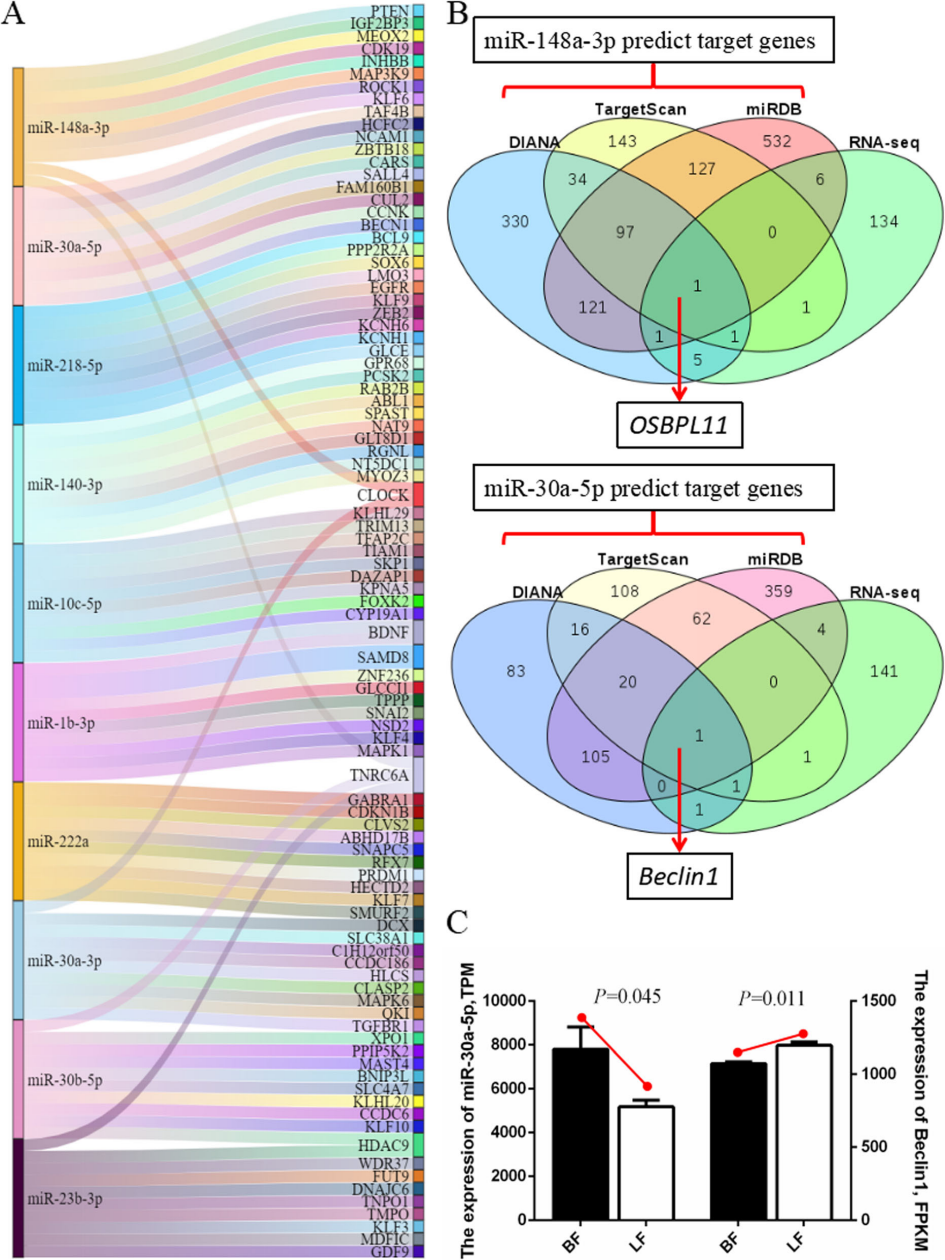
Target gene prediction of differentially expressed miRNA. (**A**) Sankey diagram of the top 10 differentially highly expressed miRNAs and their target genes. (**B**) Venn diagrams of predicted target genes of miR-148a-3p and miR-30a-5p and RNA-seq differentially expressed genes. (**C**) Comparison of expression levels of miR-30a-5p and Beclin1 in the LF and BF sequencing. Data are presented as mean ± SEM

To study the roles of the miRNAs in the development and atresia of chicken follicles, we selected the 10 highly expressed DEMs as candidates for biological function verification (Table 1). We predicted the target genes and constructed a miRNA-mRNA interaction network to identify their special biological functions (Fig. 3A). Furthermore, we predicted the target genes of the two most expressed miRNAs (miR-148a-3p and miR-30a-5p) using the bioinformatics prediction software (TargetScan, miRDB, and Diana). In addition, the same batch of miRNA-seq were subjected to mRNA transcriptome sequencing, and then the target genes were predicted by the miRNA. The genes that were differentially expressed by the RNA-seq were jointly analyzed (Accession number: PRJNA721929). The detailed gene list is shown in Additional file 6: Table S4 and Additional file 7: Table S5. The results showed that miR-148a-3p and miR-30a-5p targets OSBPL11 and Beclin1 respectively (Fig. 3B). The sequencing results of the miRNA and mRNA revealed that the expression of miR-30a-5p in the BF was higher than that in the LF, whereas Beclin1 showed a contrast trend with a higher expression of miR-30a-5p in the LF (Fig. 3C). This result was consistent with the expression pattern of the miRNA. Combined with the KEGG in Fig. 2F, the autophagy-related pathway was enriched, therefore, we selected miR-30a-5p for functional verification.

**Table 1:** Top 10 highly differentially expressed miRNAs

miRNA ID	Broody chicken follicles (mean TPM)	Laying chicken follicles (mean TPM)	log_2_ (Fold change)	*P* value	Regulation (BF/LF)
gga-miR-148a-3p	116,493.3666	148,783.0345	−0.49556	0.021398	DOWN
gga-miR-30a-5p	7793.144275	5181.34161	0.41512	0.045714	UP
gga-miR-218-5p	3030.611251	3674.221769	−0.42753	0.0060209	DOWN
gga-miR-140-3p	3446.499163	2461.166571	0.31519	0.034421	UP
gga-miR-10c-5p	2348.10433	3136.672743	−0.55872	0.044067	DOWN
gga-miR-1b-3p	323.6504074	603.2860019	−0.84955	0.040798	DOWN
gga-miR-222a	349.7456881	444.4129543	−0.48027	0.0059791	DOWN
gga-miR-30a-3p	317.8538436	180.7132371	0.63893	0.0014581	UP
gga-miR-30b-5p	331.1413545	160.2653248	0.79239	0.009194	UP
gga-miR-23b-3p	230.3833972	145.7358884	0.50257	0.00031451	UP

Regulation in BF

### Identification of chicken granulosa cells and expression of miR-30a-5p in the different tissues

**Fig. 4:**
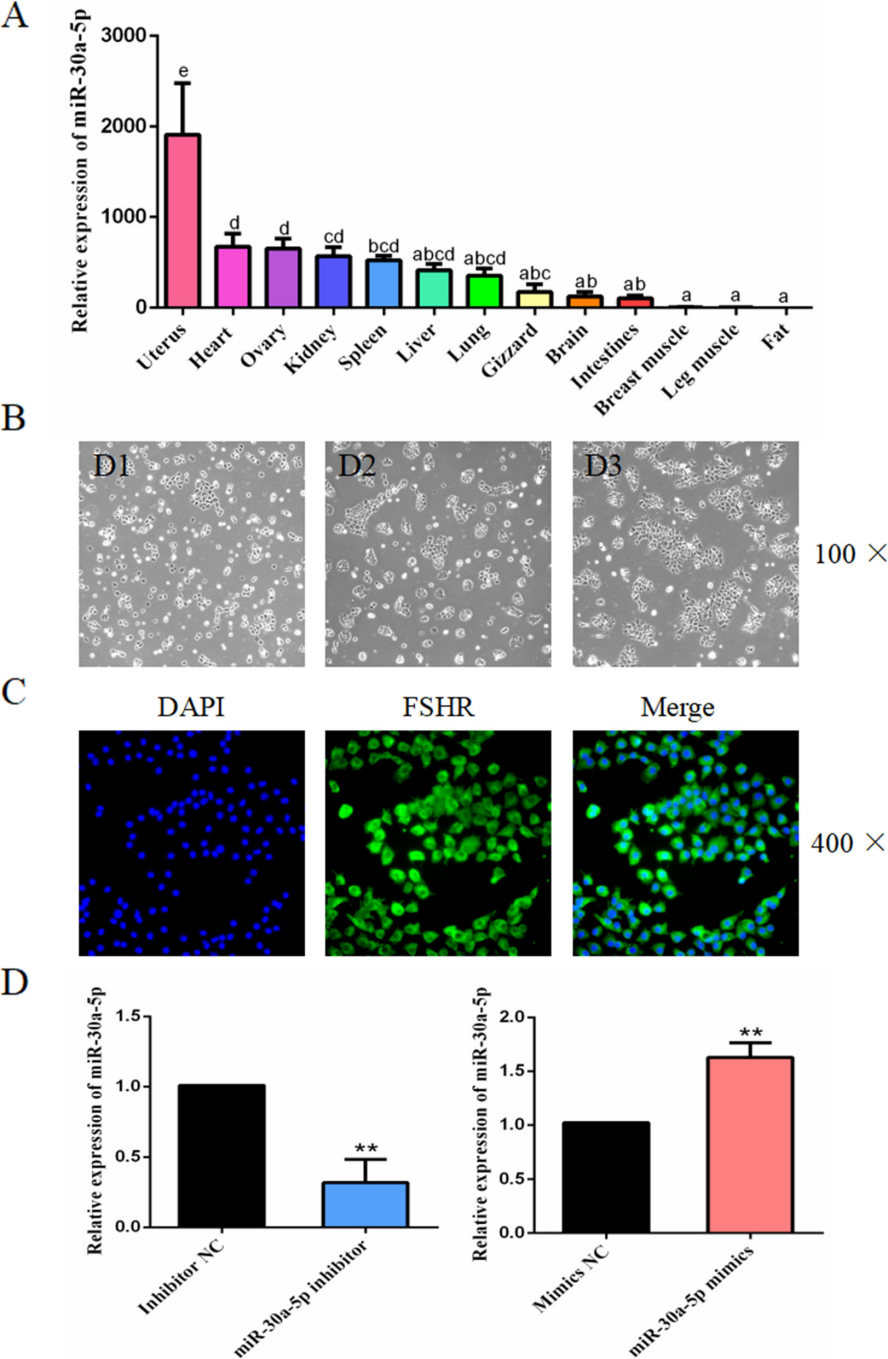
The expression of miR-30a-5p in different chicken tissues and identification of granulosa cells. (**A**) qPCR was used to detect the expression of miR-30a-5p in the 13 different tissues of chicken (fat was the control group, *n =* 9). (**B**) Morphological changes of the chicken granulosa cells at 1–6 d, 100 ×. (**C**) Representative images of the immunofluorescence staining of the FSHR at different magnifications. FSHR: green, granulosa cell marker; DAPI: blue, nucleus; Merge: identification of granulosa cells. (**D**) The expression of miR-30a-5p in the granulosa cels transfected with miR-30a-5p inhibitor or miR-30a-5p mimics. Data are presented as mean ± SEM. ^a, b, c, d^ *P* < 0.05, **P < 0.01 vs. NC

The expression levels of miR-30a-5p in the heart, liver, spleen, lung, kidney, chest muscle, leg muscle, brain, intestine, fat, gizzard, ovary, and uterus were determined, and the results showed that miR-30a-5p was highly expressed in the reproductive organs such as uterus and ovary (Fig. 4A). The primary granulosa cells were separated from the granular layer, and we found that at the initial stage, the granulosa cells were round or elliptical, whereas after 48 h culture, the cells undergo proliferation and growth having a polygonal or spindle-shaped. Furthermore, the FSHR immunofluorescence assay was performed to identify the primary granulosa cells. FSHR is a granulosa cell specific protein expressed and localized in the cytoplasm of granulosa cells [20] (Fig. 4C). In addition, the results obtained from the transfection efficiency of the miR-30a-5p inhibitor and miR-30a-5p mimics showed that the miR-30a-5p inhibitor decreased the expression of miR-30a-5p and the exogenous miR-30a-5p also increasesd the expression of miR-30a-5p (Fig. 4D).

### miR-30a-5p can target Beclin1 and inhibit its expression

**Fig. 5:**
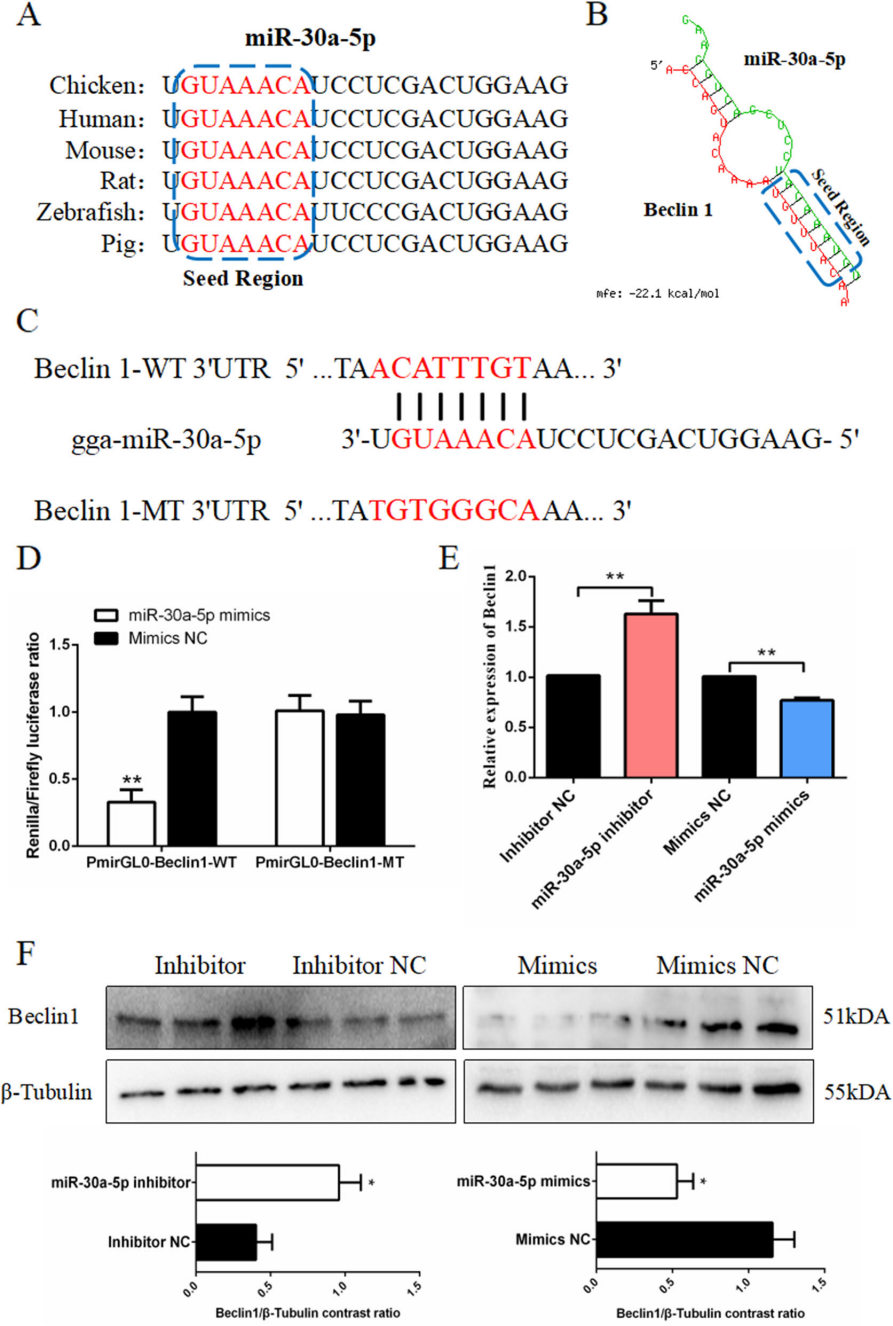
Beclin1 as a target gene of miR-30a-5p. (**A**) Comparison of miR-30a-5p seed sequences of different species. (**B**) Schematic diagram of the binding of miR-30a-5p and Beclin1. (**C**) Construction of wild-type dual-luciferase reporter gene and mutant dual-luciferase reporter gene of Beclin. (**D**) Luciferase assay is performed by co-transfecting wild-type or mutant-type dual-luciferase reporter gene with miR-30a-5p mimic or mimic NC in granulosa cells (*n =* 6). (**E**) The expression level of Beclin1 after interference or overexpression of miR-30a-5p (*n =* 9). (**F**) The protein abundance of Beclin1 after interference or overexpression of miR-30a-5p (*n =* 3). Data are presented as mean ± SEM. * *P* < 0.05, **P < 0.01 vs. NC

We predicted that miR-30a-5p might target Beclin1, so we next verified the relationship between miR-30a-5p and Beclin1. To confirm our prediction that miR-30a-5p might target Beclin1, a verification experiment was conducted to determined the relationship between miR-30a-5p and Beclin1. Seed sequence alignment identified miR-30a-5p as a widely conserved miRNA in species including human, mouse, rat, chicken, zebrafish and pig (Fig. 5A). We discovered that miR-30a-5p binds to the 3’UTR of Beclin1 through the seed sequence “GUAAACA” (Fig. 5B). Therefore, we conducted a dual luciferase reporter experiment to verify the targeting relationship between miR-30a-5p and Beclin1. We also constructed the sequence near the 3’UTR binding site of Beclin1 into the pmirGL0 vector and named it the wild-type dual luciferase reporter gene (pmirGL0-Beclin1-WT). Thereafter, we mutated the sequence of the 3’UTR binding site of Beclin1, and named it the mutant dual-luciferase reporter gene (pmirGL0-Beclin1-MT) (Fig. 5C), and then co-transfected the dual luciferase reporter gene (WT/MT) and miR-30a-5p mimics/mimics NC into the DF-1 cells. We found that the firefly luciferase activity was decreased after the pmirGLO-Beclin1-WT and miR-30a-5p mimics were co-transfected. However, the cells co-transfected with miR-30a-5p mimics and pmirGL0-Beclin1-MT remained unchanged (Fig. 5D). Moreover, after inhibiting miR-30a-5p in the granulosa cells, we discovered that the mRNA and protein expressions of Beclin1 were up-regulated. However, the mRNA and protein expressions of Beclin1 decreased after transfection of miR-30a-5p mimics (Fig. 5E and F). Taken together, these results indicate that Beclin1 is the target gene of miR-30a-5p.

### miR-30a-5p inhibits autophagy in the chicken granulosa cell

**Fig. 6:**
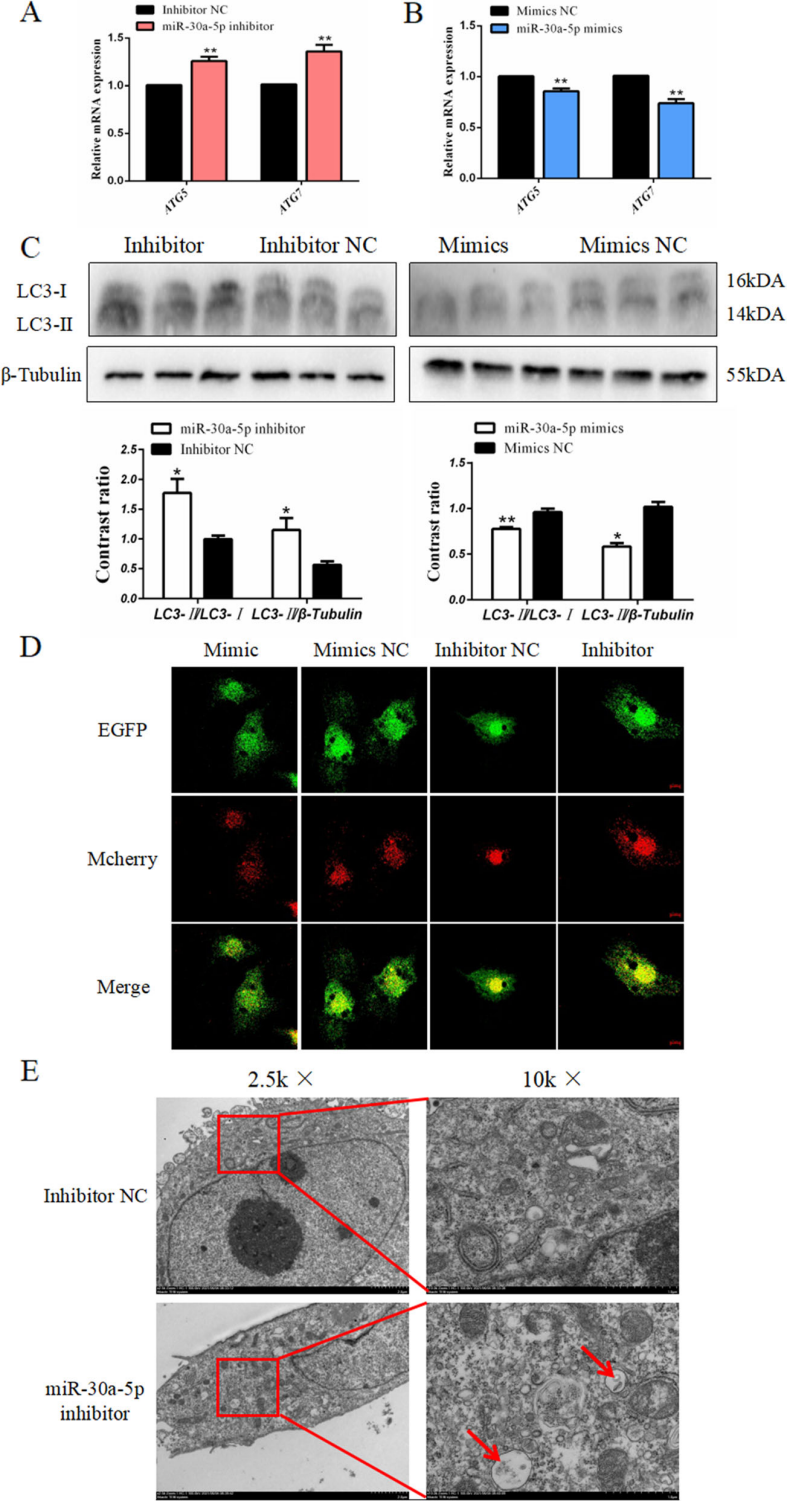
Effect of miR-30a-5p on autophagy of granulosa cells. (**A**, **B**) The expression level of *ATG5* and *ATG7* after interference or overexpression of miR-30a-5p (*n =* 9). (**C**) The protein abundance of LC3 after interference or overexpression of miR-30a-5p (*n =* 3). (**D**) Adenovirus fluorescence picture of mcherry-EGFP-LC3 after interference or overexpression of miR-30a-5p. mcherry was used for marking and tracking LC3. The weakening of EGFP can indicate the fusion of lysosomes and autophagosomes to form autophagolysosomes. The yellow spots after the merge are autophagosomes (mcherry+EGFP). (**E**) Transmission electron micrograph of granulosa cells after interference or overexpression of miR-30a-5p. The red arrow points to autophagolysosomes. Data are presented as mean ± SEM. * *P* < 0.05, **P < 0.01 vs. NC

After confirming the inhibitory role of miR-30a-5p on Beclin1, we further determined the regulatory role of miR-30a-5p in the chicken granulosa cell autophagy. Through qPCR experiments revealed that the absence of miR-30a-5p promotes the expression of autophagy marker genes autophagy related 5 (*ATG5*) and autophagy related 7 (*ATG7*) (Fig. 6A), whereas the expression of *ATG5* and *ATG7* was inhibited after the miR-30a-5p mimics were transfected (Fig. 6B). Furthermore, the protein levels of the autophagy marker protein LC3 showed a consistent trend, but after the addition of the miR-30a-5p inhibitor, the protein abundance of LC3-II was significantly increased, while the addition of exogenous miR-30a-5p knocked down the protein abundance of LC3-II (Fig. 6C). Furthermore, mcherry-EGFP-LC3 adenovirus was used to evaluate the degree of autophagy flux. As shown in Fig. 6D, the number of autophagosomes (Merge) decreased after miR-30a-5p overexpression, whereas the reduction of miR-30a-5p resulted in severe autophagosomes (Merge). The electron microscopy showed that the number of autophagosomes in the transfected miR-30a-5p inhibitor cells were increased compared to the control cells (Fig. 6E). These results indicate that miR-30a-5p showed a negative effect on the regulation of chicken granulosa cells.

### miR-30a-5p reduces apoptosis in chicken granulosa cell

**Fig. 7:**
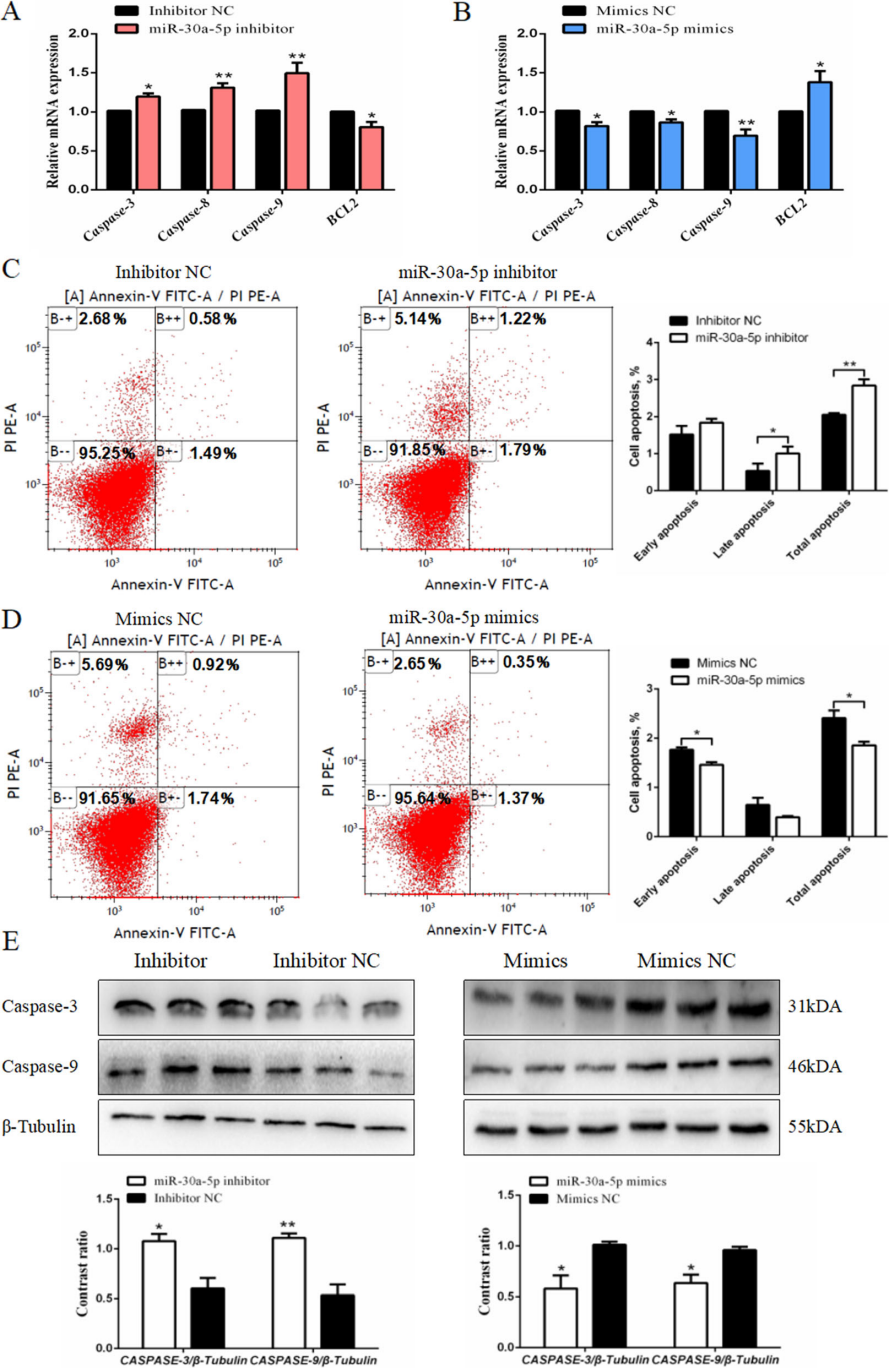
Effect of miR-30a-5p on apoptosis of granulosa cells. (**A**, **B**) The expression level of caspase-3, caspase-8, caspase-9 and *BCL2* after interference or overexpression of miR-30a-5p (*n =* 9). (**C**, **D**) Scatter plot and apoptosis rate of flow cytometry analysis after interference or overexpression of miR-30a-5p. The statistics of the early apoptosis rate, late apoptosis rate and total apoptosis rate (*n =* 3). (**E**) The protein abundance of caspase-3 and caspase-9 after interference or overexpression of miR-30a-5p (*n =* 3). Data are presented as mean ± SEM. * *P* < 0.05, **P < 0.01 vs. NC

Chicken granulosa cell apoptosis cause atresia in ovarian follicles. To investigate the effect of miR-30a-5p on chicken granulosa cell apoptosis, we measured the levels of expression of the apoptosis related marker genes, such as caspase-3, caspase-8, caspase-9 and apoptosis regulator *BCL2*. Figure 7A and B showed that miR-30a-5p negatively influenced the expression of caspase family genes and has a positive effect on the expression of *BCL2*. The transfected granulosa cells were stained with an annexin V antibody and Propidium Iodide (PI), and then the cell apoptosis was analyzed using the flow cytometry. The results showed that miR-30a-5p inhibitor promoted the apoptosis of granulosa cells, and the apoptotic rate of the granulosa cells decreased after miR-30a-5p mimics transfection (Fig. 7C and D). In addition, the protein abundance of the intrinsic apoptosis markers genes such as caspase-3 and caspase-9 were determined. Compared with the control group, the protein abundance of caspase-3 and caspase-9 increased after miR-30a-5p knocked-down. However, high expresssion of miR-30a-5p also increased the protein expression of caspase-3 and caspase-9 (Fig. 7E). These results showed that miR-30a-5p inhibits granulosa cell autophagy as well as decreased granulosa cell apoptosis.

### miR-30a-5p promotes steroid hormones synthesis in chicken granulosa cells

**Fig. 8:**
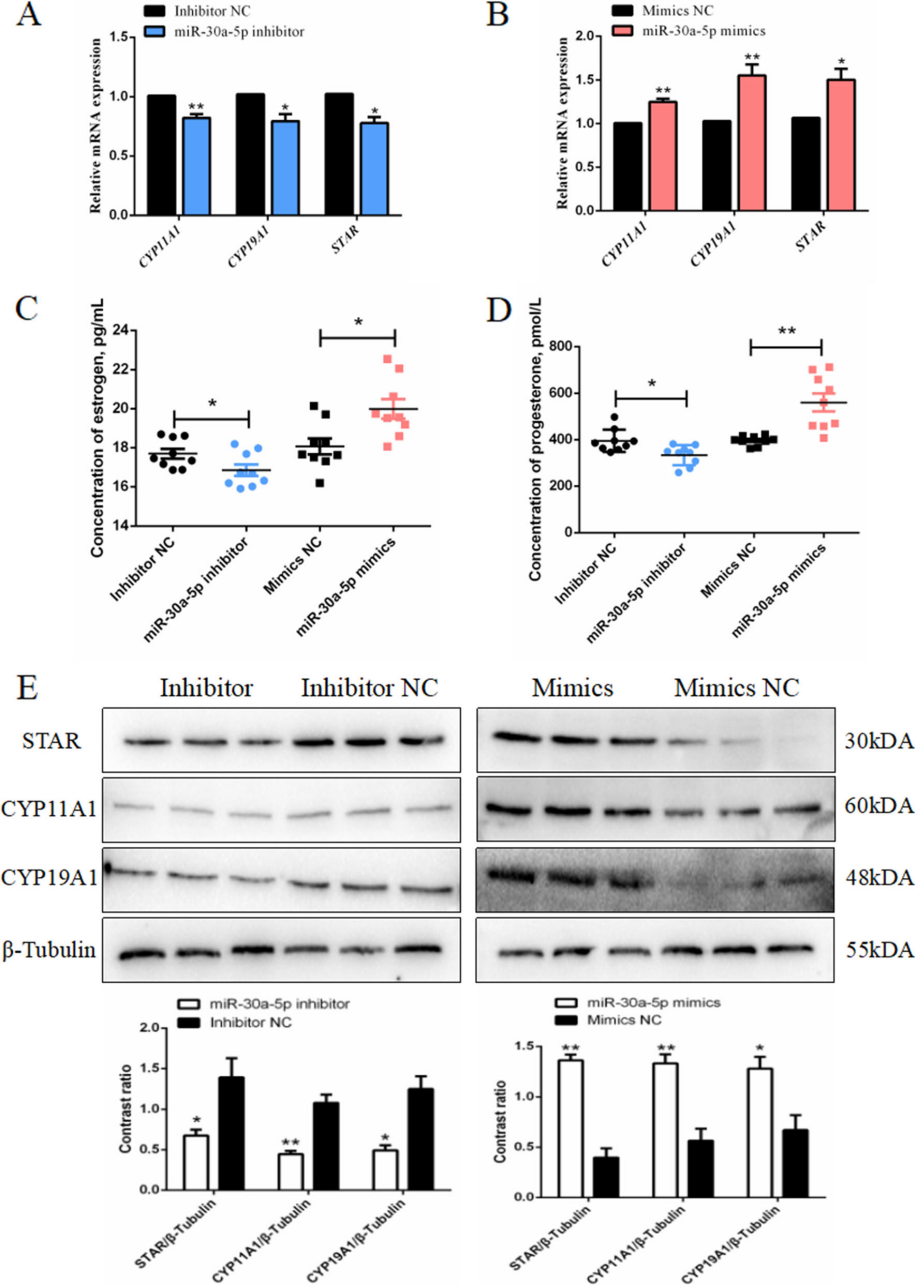
Effect of miR-30a-5p on steroid hormones synthesis of granulosa cells. (**A**, **B**) The expression levels of *CYP11A1*, *CYP19A1*, and *STAR* after the interference or overexpression of miR-30a-5p (*n =* 9). (**C**, **D**) The levels of estrogen and progesterone in the cell supernatant after interference or overexpression of miR-30a-5p (*n =* 9). (**E**) The protein expression levels of *CYP11A1*, *CYP19A1*, and *STAR* after the interference or overexpression of miR-30a-5p (*n =* 3). Data are presented as mean ± SEM. *P < 0.05, **P < 0.01 vs. NC

Granulosa cells are the main cells that synthesize steroid hormones in the follicles. In this study, we determined the effect of miR-30a-5p on the synthesis of estrogen and progesterone in the granulosa cells. The key genes cytochrome P450 family 11 subfamily A member 1 (*CYP11A1*), steroid-producing acute regulatory protein (*STAR*) and cytochrome P450 family 19 subfamily A member 1 (*CYP19A1*) related to the synthesis of E2 and P4 were determined using qPCR analysis. The results as shown in Fig. 8A and B indicated that miR-30a-5p inhibitor decreased the expression of these steroid hormone-related genes compared to the control. The cell supernatants were collected after transfection with miR-30a-5p inhibitor/miR-30a-5p mimics, and then the levels of the E2 and P4 were determined by ELISA test. Moreover, we found that overexpression of miR-30a-5p increased the levels of E2 and P4, similary, low expression of miR-30a-5p sinifcanlty decreased the levesl of E2 and P4 (Fig. 8C and D). It was confirmed through Western Blotting that miR-30a-5p promoted the synthesis of E2 and P4 (Fig. 8E). In summary, these results showed that the expression of miR-30a-5p accelerate the synthesis of steroid hormones (E2 and P4).

### miR-30a-5p reduces the mitochondrial membrane potential and promotes ROS accumulation

**Fig. 9:**
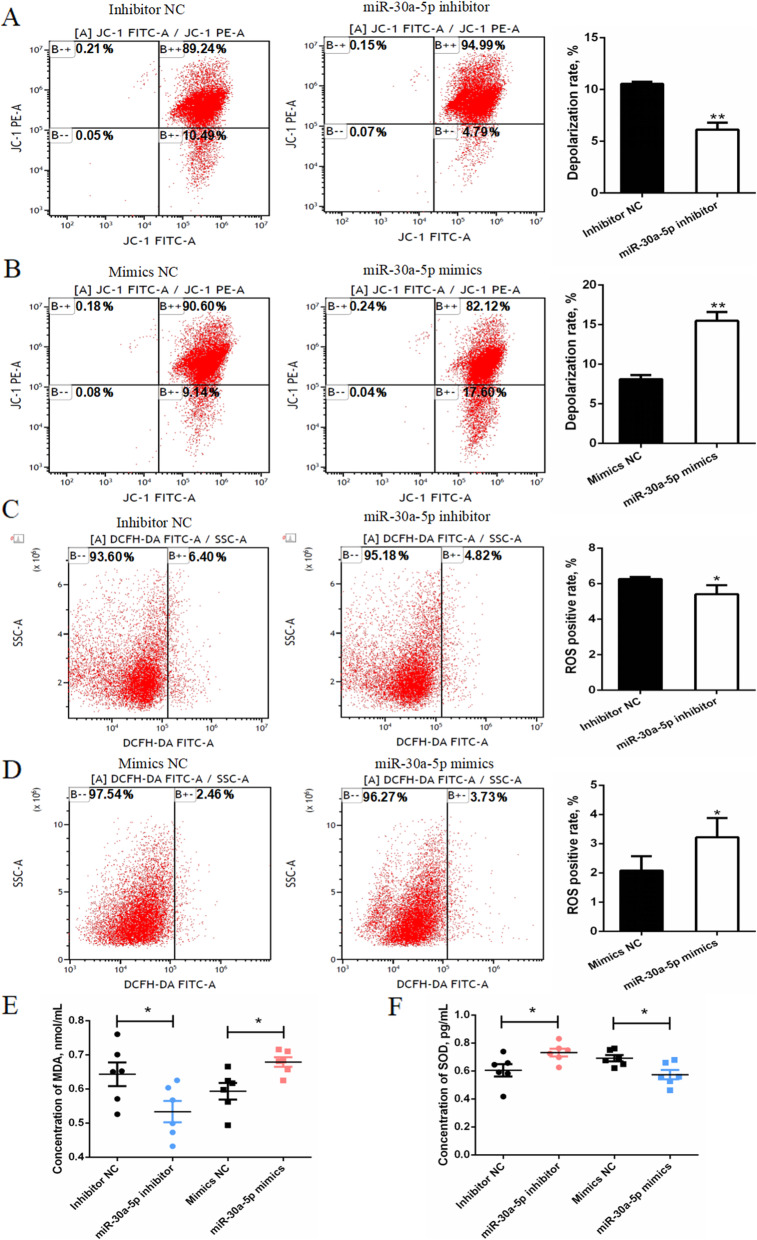
Effect of miR-30a-5p on oxidative stress of granulosa cells. (**A**, **B**) Flow cytometry analysis of mitochondrial cell membrane potential after the interference or overexpression of miR-30a-5p. The statistics of the depolarization rate of the mitochondrial membrane potential (*n =* 3). (**C**, **D**) ROS levels were analyzed using flow cytometry after the interference or overexpression of miR-30a-5p. The statistics of the number of ROS positive cells (*n =* 3). (**E**, **F**) The levels of MDA and SOD in the cell supernatant after interference or overexpression of miR-30a-5p (*n =* 6). Data are presented as mean ± SEM. * *P* < 0.05, **P < 0.01 vs. NC

The effects of miR-30a-5p on the oxidative stress in granulosa cells was determined using the flow cytometry. The results showed that the exogenous miR-30a-5p increases the depolarization rate of the mitochondrial membrane potential, however, after inhibiting miR-30a-5p, the depolarization rate of mitochondrial membrane potential decreased (Fig. 9A and B). Moreover, flow cytometry analysis showed that the accumulation or knockdown of miR-30a-5p increased or decreased the rate of ROS in the granulosa cells respectively (Fig. 9C and D). This result is consistent with the ELISA results of MDA and SOD. The accumulation of miR-30a-5p increased the content of MDA and decreased the content of SOD. Interfering with the accumulation of miR-30a-5p increased the content of SOD and decreased the content of MDA (Fig. 9E and F). These results showed that miR-30a-5p promotes oxidative stress of granulosa cells by decreasing the mitochondrial membrane potential and promoting ROS accumulation.

### miR-30a-5p regulates chicken granulosa cells autophagy and apoptosis by inhibiting Beclin1

**Fig. 10:**
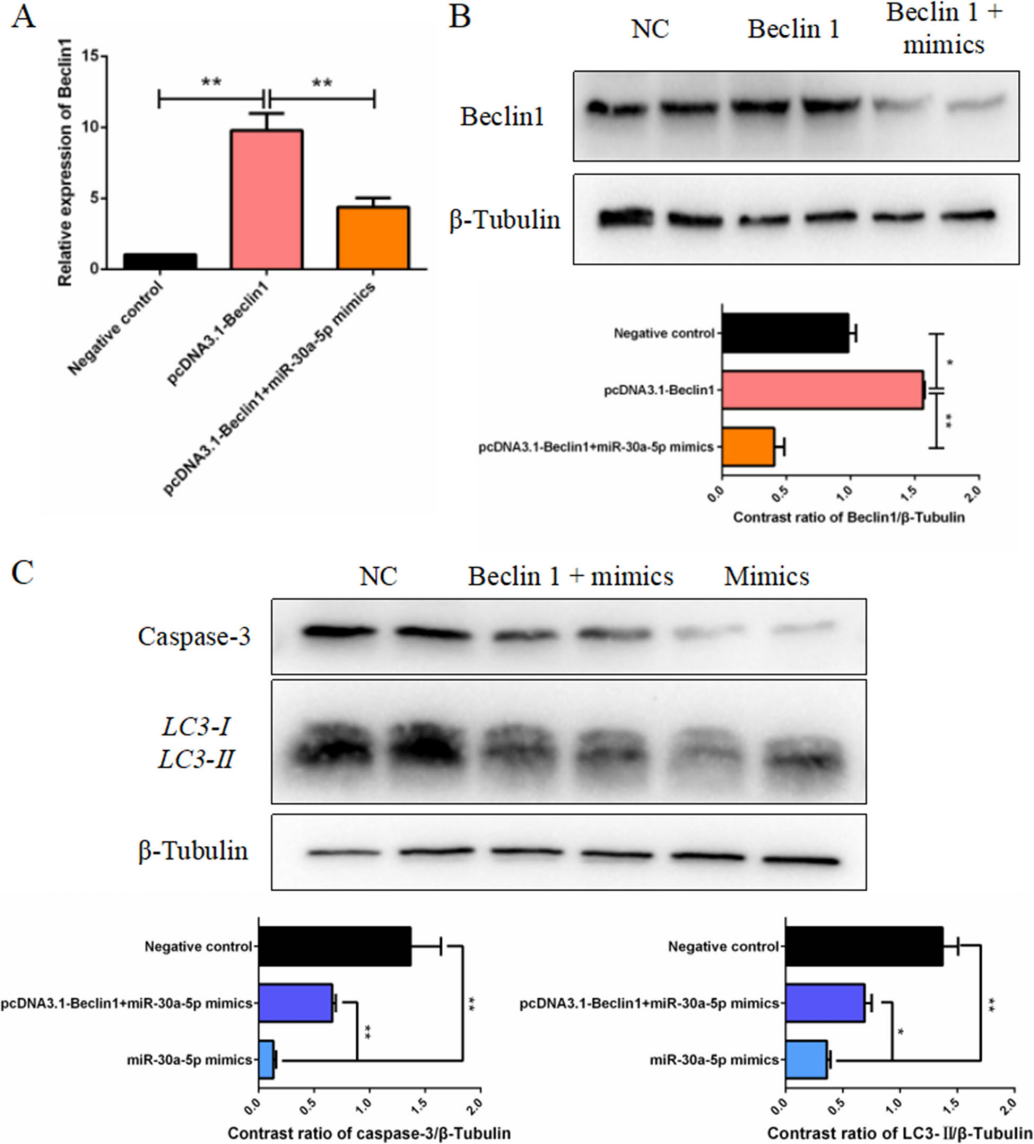
miR-30a-5p inhibits autophagy and apoptosis of granulosa cells by targeting Beclin1. (**A**) The expression levels of Beclin1 after the transfection of NC, Beclin1 overexpression plasmid, Beclin1 overexpression plasmid + miR-30a-5p mimics in the granulosa cells (*n =* 9). (**B**) The protein expression levels of Beclin1 after the transfection of NC, Beclin1 overexpression plasmid, Beclin1 overexpression plasmid + miR-30a-5p mimics in the granulosa cells (*n =* 3). (**C**) The protein expression levels of caspase-3 and LC3 after the transfection of NC, Beclin1 overexpression plasmid + miR-30a-5p mimics, miR-30a-5p mimics in the granulosa cells (*n =* 3). Data are presented as mean ± SEM. * *P* < 0.05, **P < 0.01 vs. NC

The relationship betwen the miR-30a-5p targeting Beclin1 and miR-30a-5p inhibiting autophagy and apoptosis of chicken granulosa cells was determined by inserting the coding sequence (CDS) of Beclin1 into the pcDNA3.1(+) vector to construct a Beclin1 overexpression vector. The co-transfection experiments were carried out in three sets. As shown in Fig. 10A and 10B, the results showed that the transfection of Beclin1 overexpression vector promoted the mRNA and protein expression levels of Beclin1, whereas the overexpression of Beclin1 and miR-30a-5p restored the mRNA and protein expression levels. However, the co-transfection experiment showed that the miR-30a-5p inhibited the protein expression levels of the apoptosis marker protein caspase-3 and hinder the conversion of the autophagy marker protein LC3-I to LC3-II (Fig. 10C). Therefore, these results indicated that miR-30a-5p inhibits autophagy and apoptosis of chicken granulosa cells by inhibiting Beclin1.

## Discussion

In this study, we selected healthy follicles and atretic follicles as the models for sequencing. The atretic follicles were sampled from the broody chicken. Broodiness is a behavior commonly occurring in the poultry industry, which is characterized by egg-laying cessation, inappetence and incubation. Different from laying chicken, the ovary and oviduct of broodiness chicken is degenerate. Thus, this behaviour causes shrinking of the fallopian tubes and ovaries, as well as inhibiting follicle development, promotes the appearance of atretic follicles and reduces the granular layer and membrane cells in the follicles [4, 21]. However, recent systematic and intensive breeding techniques lowers broodiness in most poultry species except in some local breeds. For instance, studies indicated that in Chinese Qingyuan (Q line) chickens the occurrence of an average broodiness is approximately 15% [22]. Therefore, in this study, we sampled the atretic follicles from Tianfu broiler breeder chickens, a local breed developed by the Chicken Breeding Unit of Sichuan Agricultural University, China.

MiRNAs are short non-coding RNAs that regulate gene expression after transcription. In the past years, human and animal studies on the ovarian miRNAs have revealed the key roles played by miRNAs in ovarian development [23]. These previous studies showed that miRNAs function mainly through granulosa cell apoptosis. Therefore, miRNAs play reguatory roles in poultry production. High-throughput transcriptome analysis is a technique used to study the sequencing of miRNAs of chicken follicles at different developmental stages. Through this sequencing techniques, miRNAs were specifically found to be involved in regulating follicular growth, maturation and atrasia by targeting matrix metalloproteinases (MMP) [24]. Kang et al. conducted an in vitro experiment and reported that the expression of miR-26a and TNRC6A in ovaries and follicles were negatively correlated, and miR-26a could combined with TNRC6A to increase the expression of BCL-2 [25].

In this study, after the deep miRNA sequencing, we found 50 differentially expressed miRNAs, among which miR-30a-5p was highly differentially expressed (the first two). Combined with the KEGG enrichment results of the sequencing data, there are obvious biological differences between healthy follicles and atretic follicles, such as differences in the process of autophagy and steroid hormone synthesis. Therefore, we hypothesized that miR-30a-5p may be a regulator of chicken follicular development and atresia, and then we also found that miR-30a-5p targeted Beclin1. The targeting relationship of miR-30a-5p to Beclin1 was verified in other cells. Studies indicated that miR-30a is down-regulated in liver fibrosis models, and its overexpression prevents liver fibrosis by inhibiting beclin1-mediated autophagy [26]. In cardiomyocytes, miR-30a can reduce the expression of Beclin-1 and LC3-II, leading to autophagy and ultimately cell damage [27]. In addition, the lncRNA-PVT1/miR-30a-5p/Beclin-1 axis is a potential target to improve severe acute pancreatitis [28]. Beclin1, a mammalian homolog of yeast *ATG6*, is an autophagy protein and tumor suppressor molecule that plays a central role in autophagy. Furthermore, Beclin1 interacts with *BCL-2* to regulate cell apoptosis. This interaction can be disrupted by phosphorylation of *BCL-2* and Beclin1, or ubiquitination of Beclin1 [29]. Interestingly, caspase-mediated Beclin1 cleavage promotes the crosstalk between apoptosis and autophagy [30]. Therefore, Beclin1 can be used as a regulatory factor to affect cell autophagy and apoptosis.

Apoptosis is a basic physiological mechanism to maintain stability in an organism. In mammals, apoptosis was reported to cause follicular atresia [5]. In poultry, follicular atresia occurs at all stages of follicular development. It mostly show a bleeding spots on the surface of the follicle, and cause shrinking and deformation of the follicles. Tilly et al. found that there was granulosa cell apoptosis in chicken follicle atresia [6]. In normal growing follicles, granulosa cell division, proliferation and apoptosis also coexist [3]. Therefore, apoptotic proportion indicates the occurrence of atresia, for instance, when the proportion of apoptotic granulosa cells in the developing follicle reaches more than 10%, then follicular atresia has occurs [7]. Johnson et al. confirmed that follicular atresia in chicken ovary *STAR*ts at the granular layer and is regulated by apoptosis [8]. However, apoptosis is not the only factor in causing follicular atresia. The induction of autophagy also mediates the growth, atresia and differentiation of follicles. A study by Choi et al. further showed that the accumulation of autophagosomes during the development and atresia of the rat ovarian follicles induce granulosa cell apoptosis by reducing the expression of *BCL2* and downstream caspase activation. This indicated that the accumulation of autophagosomes to a certain level may promote granulosa cells apoptosis [31, 32]. In our study, miR-30a-5p was differentially expressed between the healthy follicles and atretic follicles, inhibited autophagy and apoptosis of granulosa cells in vitro by targeting Beclin1. Therefore, the mechanism by which miR-30a-5p regulates chicken granulosa cell apoptosis is by inhibiting the accumulation of autophagosomes.

There are relatively few studies on the effect of autophagy on broodiness in chickens. However, in geese, hormones and autophagy are important factors that control broodiness. The broodiness in geese is related to an increase in autophagy of granulosa cells and the imbalance of homeostasis of the follicular environment [33]. Other studies indicated that the transcriptome analysis of broody Zhejiang white goose follicles enriched autophagy and hormone-related signaling pathways, the contents of progesterone and estradiol in follicles were altered, and the levels of autophagy levels in the follicles were enhanced during the broody stage [34]. In addition, ROS and autophagy played an important role in follicular development, and the interaction between the two may play a role in regulating oviposition. For instance, in geese, ROS activates the autophagy of the granulosa cells through the mTOR pathway [35]. Similar reports showed that the actvation of AKT-mediated mTOR inhibits autophagy of the granulosa cells during follicular atresia in rats [36]. Studies have revealed that melatonin mediates the survival of granulosa cells during follicular atresia by inhibiting FOXO1 [37]. Oxidative stress can induce autophagy, whereas the accumulation of autophagy reduces ROS [38]. Bellot et al. found that HIF-1 could reduce the levels of ROS through autophagy, thereby selectively removing mitochondria in cells [39]. In this study, we also found that miR-30a-5p inhibited autophagy and increase the level of oxidative stress. Similar study reported that miR-30a modulates the reduction of hypoxia/reoxygenation injury by inhibiting Beclin1-mediated autophagy, and protects elderly cardiomyocytes [40].

Steroid hormones are synthesized from cholesterol through a series of enzymatic reactions. Granulosa cells are mainly responsible for the synthesis of steroid hormones, including estrogen and progesterone. In granulosa cells, a series of enzymes are responsible for converting cholesterol into steroid hormones. Thus, emzymes such as P450scc (encoded by *CYP11A1*) catalyzes the first rate-limiting step in the conversion of cholesterol to pregnenolone, whereas cytochrome P450 aromatase (coded by *CYP19A1*) is involved in the last step of estrogen biosynthesis [41, 42]. In this study, it was revealed that miR-30a-5p promoted the synthesis of steroid hormones in the granulosa cells. However, other reports showed that the huge signal network downstream of estrogen can promote autophagy, whereas reducing over-stimulated autophagy, hence, to some extent, estrogen can regulate autophagy [43]. In conclusion, the regulatory network of the miRNA-autophagy-estrogen, involving the role of estrogen in regulating autophagy through negative feedback, requires further studies.

## Conclusion

**Fig. 11:**
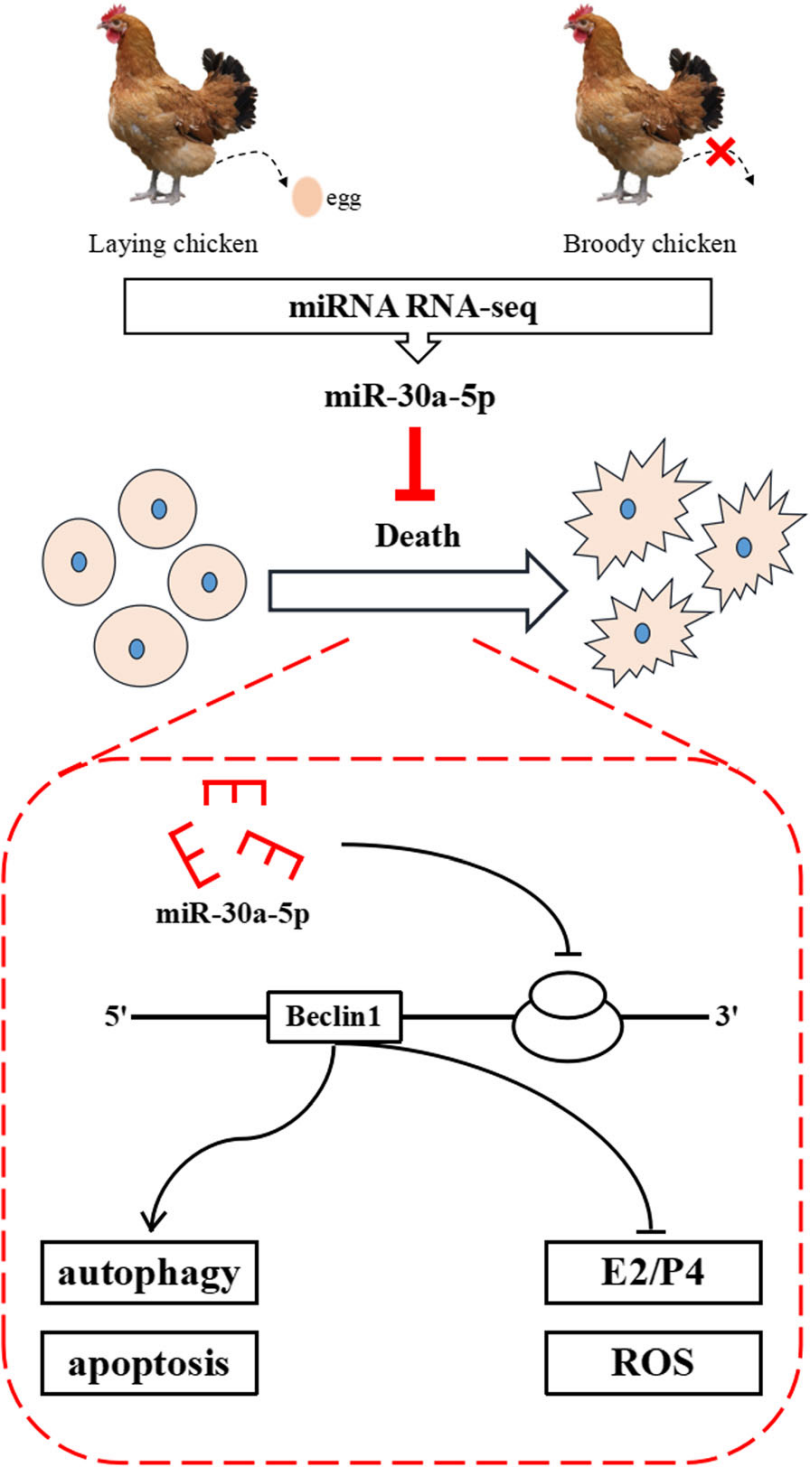
Schematic diagram of miR-30a-5p regulating chicken follicular development and atresia

In this study, we performed deep miRNA sequencing on healthy and atretic follicles. Thereafter, the highly differentially expressed miR-30a-5p from the chicken primary granulosa cells were selected for functional determination. The results showed that miR-30a-5p could inhibit the autophagy and apoptosis of granulosa cells. In addition, miR-30a-5p could promote the synthesis of steroid hormones and increase the level of oxidative stress. The dual luciferase report confirmed that Beclin1 is the target gene of miR-30a-5p. To sum up, miR-30a-5p inhibits granulosa cell death via targeting Beclin1 (Fig. 11). Previous studies on the functions of miRNAs in chicken follicle development and atresia are relatively simple. Notably, we explored the effect of granulosa cell autophagy on follicular development. Previous studies of this type have been conducted in larger birds such as geese. At the level of miRNA, the function of miR-30a-5p in regulating autophagy was further verified. The results obtained in this study will serve as a tool to understand the regulatory roles of miRNA networks, as well as clarify the molecular mechanisms regulating follicular development, atresia and its disorders, and also provides new strategies for the diagnosis and treatment of infertility and other ovarian dysfunctions.

## Supplementary Information

**Additional file 1: Fig. S1.** miRNA expression TPM density distribution map.

**Additional file 2: Fig. S2.** GO analysis of differentially expressed miRNA target genes.

**Additional file 3: Table S1.** Summary of data generated from small RNA deep sequencing.

**Additional file 4: Table S2.** Primers used for quantitative real-time PCR.

**Additional file 5: Table S3.** Antibodies used in article.

**Additional file 6: Table S4.** miR-148a-3p predict target genes.

**Additional file 7: Table S5.** miR-30a-5p predict target genes.

## Data Availability

The datasets used and analyzed during the current study are available from the corresponding author on request.

## Abbreviations

miRNAs: MicroRNAs
GCs: Granulosa cells
E2: Estradiol
P4: Progesterone
MDA: Malondialdehyde
SOD: Superoxide dismutase
ROS: Reactive oxygen species
PCOS: Polycystic ovary syndrome
HPG axis: Hypothalamus-pituitary-gonad axis
PBS: Phosphate buffered saline
m199: Medium 199
FBS: Fetal bovine serum
NC: Negative control
HRP: Horseradish peroxidase
SEM: Standard error of mean
LH: Luteinizing hormone
FSH: Follicle-stimulating hormone
TPM: Transcripts per million
DEMs: Differentially expressed miRNAs
ATG5: Autophagy related 5
ATG7: Autophagy related 7
PI: Propidium iodide
CYP11A1: Cytochrome P450 family 11 subfamily A member 1
CYP19A1: Cytochrome P450 family 19 subfamily A member 1
STAR: Steroid-producing acute regulatory protein
CDS: Coding sequence
MMP: Matrix metalloproteinases
