# Supplementary figures and images for: miRNA sequencing analysis of healthy and atretic follicles of chickens revealed that miR-30a-5p inhibits granulosa cell death via targeting Beclin1
Source: J Anim Sci Biotechnol. 2022 Apr 12;13:55. doi: 10.1186/s40104-022-00697-0 (PMC9003977; doi:10.1186/s40104-022-00697-0)

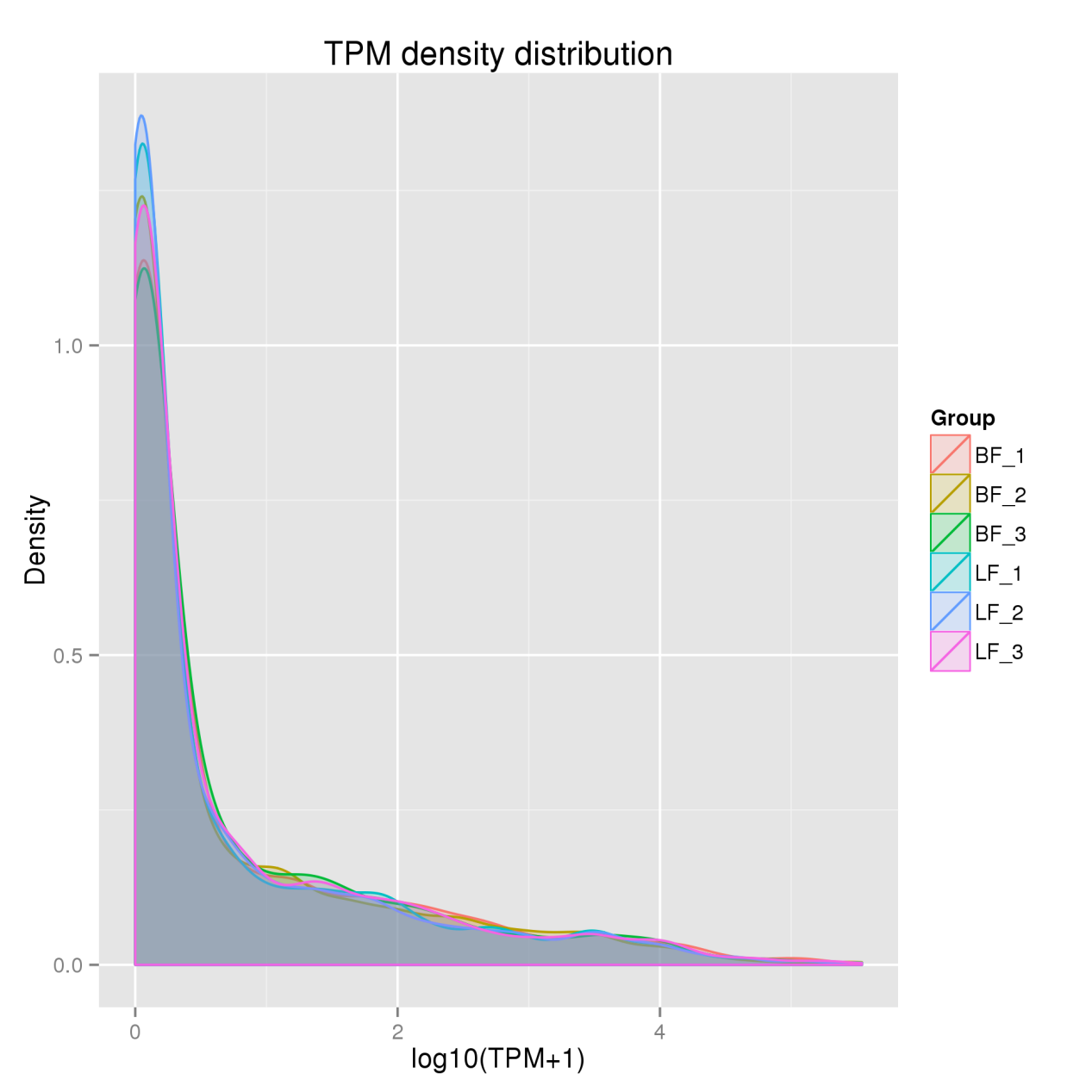


**Figure S1. miRNA expression TPM density distribution map.**

Supplement: Supplementary file 1 — Additional file 1: Fig. S1. miRNA expression TPM density distribution map. [file 40104_2022_697_MOESM1_ESM.docx]

**
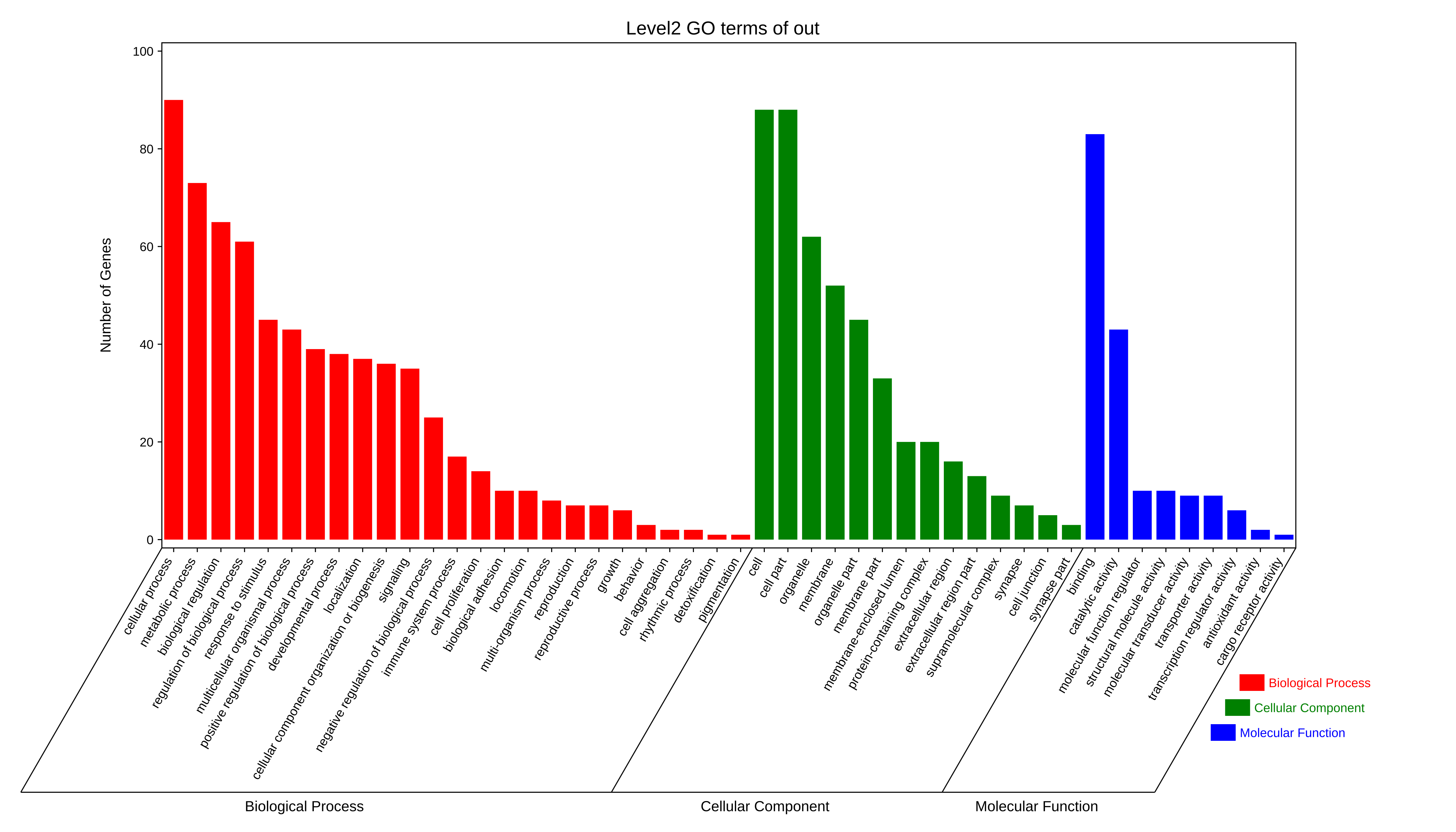
**

**Figure S2. GO analysis of differentially expressed miRNA target genes.**

Supplement: Supplementary file 2 — Additional file 2: Fig. S2. GO analysis of differentially expressed miRNA target genes. [file 40104_2022_697_MOESM2_ESM.docx]
